# Do youth e-cigarette users perceive smoking as attractive? The dynamics of demographics and contextual factors

**DOI:** 10.3389/ijph.2026.1609361

**Published:** 2026-05-26

**Authors:** Omar B. Da’ar, Maria Alamr, Randah Alalweet, Bandar AlEissa, Farah Kalmey

**Affiliations:** 1 Department of Health Systems Management, College of Public Health & Health Informatics, King Saud Bin Abdulaziz University for Health Sciences, Riyadh, Saudi Arabia; 2 King Abdullah International Medical Research Center (KAIMRC), Riyadh, Saudi Arabia; 3 Planning and Development, Ministry of Health, Riyadh, Saudi Arabia; 4 Population Health, Ministry of Health, Riyadh, Saudi Arabia; 5 College of Science and Health Professions, King Saud Bin Abdulaziz University for Health Sciences, Ministry of National Guard, Riyadh, Saudi Arabia

**Keywords:** e-cigarette use, perception of smoking, Saudi Arabia, tobacco marketing, youth smoking

## Abstract

**Objective:**

This study examines whether youth e-cigarette users perceive smoking as attractive and how comfort-based norms, industry promotion, and media or environmental exposures shape these perceptions.

**Methods:**

Using a nation-wide representative 2022 Global Youth Tobacco Survey data from Saudi Arabia, we analyzed e-cigarette users’ perceptions of smoking attractiveness with descriptive statistics and a multivariable regression model to identify associated covariates.

**Results:**

Among 5,455 students aged 13–15 years, 14.3% had ever used e-cigarettes, and 26% of users viewed smoking as attractive. More males rated smoking favorably in crude analyses. However, adjusted model showed males had lower odds versus females. Youth exposed to secondhand smoke in enclosed public places, point-of-sale marketing, anti-smoking messages, and counter-branding marketing had reduced odds of viewing smoking as attractive, whereas those who found it as socially comfortable, offered free tobacco products by a company representative, or owning branded items had higher odds. Secondhand smoke at school or home was not significant.

**Conclusion:**

Findings suggest prevention may benefit from targeting comfort-based norms and industry promotion, and future research should examine causal effects to guide youth-focused policy.

## Introduction

Globally, smoking initiation most often occurs in adolescence, a period of heightened susceptibility to peer influence and media portrayals of smoking [1, 2]. The social environment, particularly peer networks, strongly shapes smoking behaviors, with young people adopting smoking patterns modeled by friends or reinforced by marketing that glamorizes tobacco use [2, 3]. Youth smoking remains a pressing public health concern, amplified by rapidly changing social norms and aggressive marketing and media promotion of vaping and other nicotine products [4, 5]. In this context, youth e-cigarette use often coincides with established conventional smoking, with social acceptance and beliefs that vaping is less harmful than cigarettes fueling these behaviors [6–8]. This perceived acceptability reinforces the idea of e-cigarettes as a preferable alternative, further increasing their appeal among young people [9].

The prevalence of e-cigarette use is strongly linked to industry marketing, underscoring the influence of advertising and promotional activities on youth vaping [10]. The e-cigarette sector is closely tied to the tobacco industry, with evidence suggesting a symbiotic relationship between e-cigarette use and youth initiation of conventional smoking that may ultimately facilitate progression to adult smoking [11–13]. Studies showed that e-cigarette marketing frequently targets young people through appealing flavors and lifestyle-oriented branding and that perceptions of vaping as less harmful than cigarettes can unintentionally encourage adolescents to experiment with both products [14, 15]. Furthermore, evidence shows that celebratory or social contexts in which e-cigarettes and conventional cigarettes co-occur can reinforce the idea that smoking signifies enjoyment and social acceptance, particularly among male adolescents [16].

Saudi Arabia’s e-cigarette market has expanded, shaping vaping as an acceptable and supposedly less harmful alternative to conventional cigarette smoking among youth [17]. While prior studies have demonstrated the prevalence of e-cigarette use in Saudi Arabia [15], the determinants of pro-smoking perceptions among young e-cigarette users remain poorly understood. This study examines whether young e-cigarette users perceive smoking as appealing and identifies the contextual factors that shape these perceptions. At the local level, it provides context-specific insights essential for designing targeted, culturally relevant prevention policies and communication strategies. More broadly, understanding these perceptions is crucial for informing public health initiatives aimed at preventing the rising burden of smoking among youth.

## Methods

### Data and study population

This study analyzed a de-identified secondary dataset from the 2022 Global Youth Tobacco Survey (GYTS) in Saudi Arabia, provided by the Ministry of Health in collaboration with the World Health Organization (WHO). The GYTS is a nationally representative, school-based survey of intermediate school students that uses the standardized WHO/CDC GYTS protocol [18]. The survey was implemented jointly by the Ministry of Health and the Ministry of Education. All intermediate students were eligible and completed an anonymous, self-administered, scannable questionnaire covering tobacco use, secondhand smoke, cessation, access, marketing exposure, and related knowledge and perceptions. Using a two-stage cluster sampling design, with schools selected proportional to enrollment size, the survey achieved an overall response rate of about 92%, yielding a final sample of 5,610 students aged 13–15 years. Scientific and ethical approvals were obtained from King Abdullah International Medical Research Center (KAIMRC) and the Ministry of Health.

### Outcome variable and covariates of interest

Our primary outcome was perceiving smoking as attractive among youth who have ever used e-cigarettes. First, we captured self-reported e-cigarette use, defined as a “*yes”* response to the question, “Have you ever used electronic cigarettes?” Among those reporting e-cigarette use, we then restricted the analysis to this subsample and categorized respondents into two groups based on whether they perceived smoking as attractive, using the item, “Do you think that young people who smoke are more attractive?” (yes/no). Finally, we examined a range of covariates associated with perceiving smoking as attractive, including demographics (age, gender, and grade level), pleasure-related factors (comfort and enjoyment), media and other exposures, tobacco marketing and branding, and exposure to anti-tobacco messages in the media. Further details on these variables are provided in Tables 1–3.

**TABLE 1:** Perception of smoking as attractive by demographics and pleasure (Saudi Arabia, 2022).

Variables	​	Attractiveness of smoking			​
​	​	Yes	No	Total	p-value
		n (%)	n (%)	n (%)	
*Demographics*					
Age	13-year-olds	57 (29.7)	166 (28.3)	223 (28.6)	0.692
	14-year-olds	70 (36.5)	202 (34.4)	272 (34.9)	
	15-year-olds	65 (33.8)	219 (37.3)	284 (36.5)	
Gender	Male	124 (65.3)	314 (53.9)	438 (56.7)	0.007*
	Female	66 (34.7)	269 (46.1)	335 (43.3)	
Grade	1st intermediate	57 (30.5)	149 (25.6)	206 (26.8)	0.362
	2nd intermediate	64 (34.2)	201 (34.5)	265 (34.4)	
	3rd intermediate	66 (35.3)	232 (39.9)	298 (38.8)	
*Comfortability and enjoyment*					
Smoking tobacco helps feel more comfortable at social gatherings	Yes	121 (66.1)	197 (34.3)	318 (41.9)	<0.001*
	No	62 (33.9)	378 (65.7)	440 (58.1)	
Thought they might enjoy smoking a cigarette	Yes	58 (75.3)	64 (24.7)	122 (36.3)	<0.001*
	No	19 (24.7)	195 (75.3)	214 (63.7)	

* p < 0.05 considered statistically significant.

### Statistical analysis

We summarized the distribution of the outcome variable (perceiving smoking as attractive vs. unattractive) and all candidate covariates using descriptive statistics. Covariates included demographic characteristics (age, gender, grade level), measures of pleasure (comfort, enjoyment), media and other exposure variables, tobacco marketing and branding, and exposure to anti-tobacco messages in the media. We first assessed crude associations between perceiving smoking as attractive and each covariate using chi-square tests for categorical variables. We then fitted a multivariate logistic regression model to identify contextual factors independently associated with perceiving smoking as attractive and to estimate adjusted odds ratios and predicted probabilities. Statistical significance was set at α = 0.05, and analyses were conducted using SPSS version 20 and cross-checked with STATA version 12.

## Results

### Proportion of e-cigarette users

**FIGURE 1:**
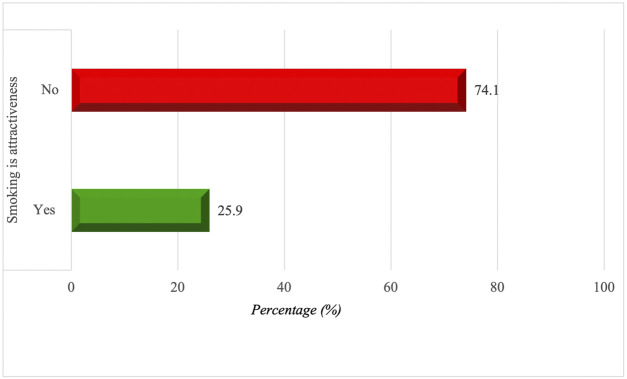
Distribution of electronic cigarette users among youth aged 13–15 years by smoking perception (Saudi Arabia, 2022).

Of the 5,455 students aged 13–15 years who participated in the 2022 GYTS, 14.3% (n = 779) reported ever using e-cigarettes. As shown in Figure 1, about one quarter of these e-cigarette users (26%, n = 202) perceived smoking as generally attractive.

Table 1 through Table 3 present the results of a Chi-Square Test regarding the association between the perception of smoking as attractive and various covariates, including demographics, comfort and enjoyment, media and other exposures, tobacco marketing and branding, and anti-tobacco messages in the media.

### Gender disparity

The association showed a clear gender gap in how youth e-cigarette users viewed smoking. Boys were almost twice as likely to see smoking as attractive (65.3%, n = 124) compared with girls (34.7%, n = 66), suggesting that, other factors being held constant, girls were much less inclined to view smoking positively than their male counterparts.

### Comfort and enjoyment

The results indicated a significant association between the perceived pleasure of smoking and its attractiveness. Among the youth who found smoking attractive, 75.3% (n = 58) held the belief that they would enjoy the experience. Conversely, among youth who viewed smoking unattractive, 75.3% (n = 195) did not believe they would enjoy the behavior. Thus, this result highlights a relationship in which expectation and perception mirror each other (Table 1).

### Media and secondhand smoke exposures

Seeing smoking on TV showed a negative association with attractiveness. Only 35.3% (n = 67) of the youth who saw smoking on TV found it attractive, compared to 64.7% (n = 123) of the youth who viewed smoking as attractive and reported no such TV exposure. Nearly half (48.6%, n = 280) of those who saw smoking on TV viewed it as unattractive (Table 2).

**TABLE 2:** Perception of smoking as attractive by media, secondhand smoke, and marketing exposures (Saudi Arabia, 2022).

Variables		Attractiveness of smoking			
		Yes n (%)	No n (%)	Total n (%)	p-value
*Media exposure*					
Saw someone using tobacco on television, videos, or movies in the past 30 days					
Yes		67 (35.3)	280 (48.6)	347 (45.3)	0.001*
No		123 (64.7)	296 (51.4)	419 (54.7)	​
Watched someone using tobacco on TV, videos, or movies					
Yes	Yes	67 (64.4)	280 (61.3)	347 (61.9)	0.578
No	No	37 (35.6)	177 (38.7)	214 (38.1)	​
*Secondhand smoke exposures*					
Have one or more parents who smoke					
Yes		50 (26.2)	145 (24.9)	195 (25.2)	0.773
No		141 (73.8)	438 (75.1)	579 (74.8)	​
Exposed to tobacco smoke at home in the past 7 days					
Yes		52 (27.5)	242 (41.9)	294 (38.3)	<0.001*
No		137 (72.5)	336 (58.1)	473 (61.7)	​
Exposed to tobacco smoke in enclosed public places in the past 7 days					
Yes		82 (43.2)	278 (48.1)	360 (46.9)	0.242
No		108 (56.8)	300 (51.9)	408 (53.1)	​
Exposed to tobacco smoke at outdoor public places in the past 7 days					
Yes		66 (34.7)	292 (50.4)	358 (46.6)	<0.001*
No		124 (65.3)	287 (49.6)	411 (53.4)	​
Saw anyone smoking inside the school building/outside school property in the past 30 days					
Yes		104 (56.2)	180 (31.7)	284 (37.7)	<0.001*
No		81 (43.8)	388 (68.3)	469 (62.3)	​
*Marketing exposures*					
Saw tobacco marketing at points of sale in the past 30 days					
Yes		38 (20.0)	131 (22.8)	169 (22.1)	0.480
No		152 (80.0)	444 (77.2)	596 (77.9)	​
Visited points of sale and saw any tobacco marketing at the points of sale in the past 30 days					
Yes		38 (40.0)	131 (33.1)	169 (34.4)	0.229
No		57 (60.0)	265 (66.9)	322 (65.6)	​

* p < 0.05 considered statistically significant.

Exposure to secondhand smoke in schools appeared to be associated with smoking perceptions. More than half (56.2%, n = 104) of those who saw people smoking in or around school buildings reported smoking as attractive, while over two-thirds (68.3%, n = 388) of those who did not see smoking at school said smoking was not attractive. Surprisingly, seeing people smoke in outdoor public places was associated with lower smoking appeal. Nearly a third (34.7%, n = 66) of the exposed youth viewed smoking as attractive, while 50.4% (n = 292) in this group found it unattractive. Yet 65.3% (n = 124) of youth who reported no outdoor exposure still believed smoking was attractive (Table 2).

### Tobacco marketing and branding

Table 3 highlights how marketing, branding, and anti-smoking messages relate to how youth viewed smoking. The chi-square results pointed to a strong association between tobacco marketing/branding and finding smoking attractive. Young people who thought smoking was attractive were more likely to wear branded tobacco gear, more likely to own an item with a tobacco logo, and more likely to have been offered free tobacco. For example, 41.6% (n = 77) of those offered free tobacco found smoking attractive, compared with only 11% (n = 62) of those who were not. Similarly, 47% (n = 85) of those who owned a branded item saw smoking as attractive, while 85.1% (n = 457) of those without such items viewed smoking as unattractive. The pattern was strongest among youth wearing or owning branded products, where 60% (n = 33) of those who viewed smoking as attractive reported having such gear, versus 35.3% (n = 83) of those who considered smoking unattractive.

**TABLE 3:** Perception of smoking as attractive by marketing and ant-tobacco messages (Saudi Arabia, 2022).

Variables	Attractiveness of smoking			
	Yes n (%)	No n (%)	Total n (%)	P-value
Marketing exposures				
Ever offered a free tobacco product from a tobacco company representative				
Yes	77 (41.6)	62 (11.0)	139 (18.6)	<0.001*
No	108 (58.4)	501 (89.0)	609 (81.4)	​
Had something with a tobacco product brand logo on it				
Yes	85 (47.0)	80 (14.9)	165 (23.0)	<0.001*
No	96 (53.0)	457 (85.1)	553 (77.0)	​
Had brand logo or wear tobacco company or product name or picture on it				
Yes	33 (60.0)	83 (35.3)	116 (40.0)	0.001*
No	22 (40.0)	152 (64.7)	174 (60.0)	​
Anit-tobacco messages				
Saw or heard anti-tobacco messages in the media in the past 30 days				
Yes	114 (62.6)	256 (45.9)	370 (50.0)	<0.001*
No	68 (37.4)	302 (54.1)	370 (50.0)	​
Saw/heard any anti-tobacco media messages at sporting or other community events in the past 30 days				
Yes	41 (21.4)	117 (20.3)	158 (20.6)	​
No	151 (78.6)	459 (79.7)	610 (79.4)	0.758
Attended sporting or other community events in the past 30 days who saw or heard any anti-tobacco messages at the events				
Yes	41 (51.3)	117 (39.3)	158 (41.8)	0.057
No	39 (48.8)	181 (60.7)	220 (58.2)	​

* p < 0.05 considered statistically significant.

### Anti-tobacco messages in the media

Table 3 illustrates a clear association between youth e-cigarette users’ perceptions of smoking and media messaging. Nearly two-thirds (62.6%, n = 114) of youth who viewed smoking as appealing reported having seen anti-tobacco messaging, indicating that these campaigns were reaching even youth who had favorable opinions on smoking. In contrast, perceptions among youth who considered smoking unattractive were more mixed, with just over half (54.1%, n = 302) reporting they had not seen recent anti-tobacco messages.

### Multivariate analysis

The multivariate analysis shown in Table 4 adjusted for such factors as gender, pleasure, media and other exposures, marketing and branding, and anti-tobacco messages. The results suggested that male e-cigarette users had 63% lower odds of perceiving smoking in general as attractive compared to females (OR = 0.37, CI: 0.20–0.69, p = 0.002). In addition, youth exposed to smoking in enclosed public places had 60% lower odds of seeing smoking as attractive compared to those who were not exposed (OR = 0.40, CI: 0.17–0.92, p = 0.031). Moreover, youth exposed to tobacco marketing at points of sale had 55% lower odds of perceiving smoking in general as attractive compared to those not exposed to such marketing (OR = 0.45, CI: 0.22–0.91, p = 0.026). Furthermore, youth with direct exposure to anti-tobacco cues and counter-branding had markedly lower odds of perceiving smoking as attractive. The results revealed that youth who reported owning or wearing items displaying a tobacco brand logo or company name had 67% lower odds of viewing smoking as attractive (OR = 0.33, CI: 0.12–0.92, p = 0.034). Similarly, youth who had seen or heard anti-smoking messages in the media had 69% lower odds of perceiving smoking as attractive (OR = 0.31, CI: 0.15–0.66, p = 0.002). On the other hand, the results revealed that young e-cigarette users who described smoking as comfortable were twice as likely to have a positive perception about smoking (OR = 2.38, CI: 1.00–5.63, p = 0.049). Similarly, young e-cigarette users who were offered a free tobacco product by a company representative had higher odds of perceiving smoking as attractive (OR = 7.88, CI: 2.01–30.91, p = 0.003). Finally, young e-cigarette users who owned an item displaying a tobacco product brand logo had higher odds of perceiving smoking as attractive (OR = 4.08, CI: 1.09–15.23, p = 0.037).

**TABLE 4:** Adjusted odds of perceiving smoking as attractive among youth electronic cigarette users (Saudi Arabia, 2022).

Variables	Odds ratio	95% CI		p-value
Male	0.368	0.196	0.693	0.002*
Smoking helps people feel more comfortable at celebrations, parties, and social gatherings	2.376	1.003	5.628	0.049*
Smoking is enjoyable	2.005	0.809	4.97	0.133
Exposed to tobacco smoke at home	0.426	0.163	1.111	0.081
Exposed to tobacco smoke in enclosed public places	0.398	0.172	0.921	0.031*
Saw anyone smoking inside the school building/outside school property	0.993	0.395	2.494	0.988
Saw tobacco marketing at points of sale	0.451	0.223	0.911	0.026*
Ever offered a free tobacco product from a tobacco company representative	7.879	2.008	30.911	0.003*
Had something with a tobacco product brand logo on it	4.078	1.092	15.229	0.037*
Had brand logo or wear tobacco company or product name or picture on it	0.325	0.115	0.920	0.034*
Saw or heard anti-smoking messages in the media	0.313	0.149	0.656	0.002*

* p < 0.05 considered statistically significant.; Gender (Female is reference); the rest of variables, “No” is the reference category.

Together, these results showed that feeling comfortable with smoking and experiencing high exposure to tobacco marketing were associated with positive views on smoking. However, the multivariate analysis revealed that enjoyability and exposure to secondhand smoke in school buildings or at home were not associated with smoking perceptions.

## Discussion

We assessed the association between the perception of smoking among youth who used e-cigarettes and covariates such as gender, pleasure, media and other exposures, marketing and branding, and anti-tobacco messages. In Chi-square analysis, we observed a clear gender difference in how youth e-cigarette users perceived the attractiveness of smoking, with more boys than females describing smoking as appealing. For example, a study of French high school e-cigarette users found that most perceived e-cigarette use as harmful, with negative views stronger among females than males [19]. Another study reported that people tended to see male smokers as more impulsive, aggressive, and potentially violent toward women, suggesting that, for many females, smoking signals undesirable traits rather than attractiveness. At the same time, male smokers were also viewed as more confident, better leaders, and more desirable for short-term relationships [20]. A further study showed that smokers rated other smokers as attractive and not less intelligent, while non-smokers viewed smokers as less attractive and less intelligent, with broad agreement between men and women [21]. Additional evidence indicates that this gender gap extends to behavior and perceptions: adolescent boys were more likely than girls to experiment with smoking and other substances [22, 23], reflecting boyhood norms that link risk-taking and smoking with success, intelligence, and masculine appeal [24].

Therefore, the crude finding that more male e-cigarette users than females perceive smoking as attractive is consistent with prior Saudi research showing stronger pro-smoking imagery among boys [25]. However, in the multivariate analysis adjusting for exposure to media advertising, free product offers, and secondhand smoke in enclosed public places, males were less likely than females to rate smoking as attractive compared to females. This suggests that apparent gender differences in pro-smoking perceptions among e-cigarette users may be confounded and potentially modified by differential exposure to pro-tobacco environments. Broader social and cultural forces may therefore shape smoking perceptions and behaviors in gender-specific ways [26], in line with evidence that smoking attitudes vary with co-occurring influences and gendered social contexts [27, 28]. Additionally, several studies indicate that in environments with strong anti-smoking messages and supportive norms, girls may be more likely than boys to reject smoking, while boys may remain less swayed by media portrayals of smoking as attractive once these other influences are accounted for [29, 30].

Our results also indicated that young e-cigarette users who believed that smoking helps people feel more comfortable at celebrations, parties, and social gatherings were more likely to have a positive view about smoking. This aligns with prior evidence that youth often use e-cigarettes and cigarettes for pleasure, autonomy, and social connection [19]. Prior studies report that teenagers frequently associate smokers with enjoyment and independence, while viewing non-smokers as more traditional and oriented toward religion, family, and social harmony [31]. Smoking can also structure peer networks, with teenagers more likely to befriend other smokers when smoking is common in their environment [32], reinforcing its image, particularly in settings such as parties and gatherings, as a socially rewarding behavior [24]. These perceptions are continually reinforced by social norms and peer influence, especially among young adults, strengthening the link between smoking, relaxation, and socializing [33]. These findings suggest that e-cigarette use may strengthen social images of smoking as a route to feeling relaxed and accepted at gatherings, consistent with qualitative studies linking vaping to “coolness” and social belonging among youth [34]. Therefore, prevention should directly counter pro-social narratives around both smoking and vaping.

Moreover, our study found that e-cigarette users exposed to secondhand smoke in public places were more likely to view smokers as unattractive rather than being attractive. This aligns with evidence that secondhand smoke exposure shapes attitudes and behaviors in a negative direction and is recognized as a serious health hazard. Whether in public places or at home, exposure is linked to heightened stress for both smokers and non-smokers [35–40]. In particular, secondhand smoke has been linked to elevated stress and depressive symptoms [36], increased risk of chronic kidney disease and reduced lung function [37, 38], and higher rates of cardiovascular diseases [39, 40]. Public awareness of the harm of secondhand smoke can shift perceptions and underscore its serious impact on health. Comprehensive smoke-free laws and related public initiatives reduce secondhand smoke exposure and lower smoking prevalence, decreasing acute coronary events, demonstrating their effectiveness in changing both behavior and perceptions [41, 42].

Furthermore, our findings indicated that e-cigarette users who were exposed to tobacco marketing, as reflected in having received free tobacco products and owning branded items, were significantly more likely to regard smoking as attractive. This is consistent with evidence that youth who viewed smoking appealing were more susceptible and receptive to tobacco promotion, which in turn increases experimentation, initiation, and continued use [43–46]. Flavored e-cigarettes are particularly enticing [47] to young people, and exposure to on-screen characters can heighten urges to smoke, amplifying the impact of marketing and media [48]. Therefore, our results align with recent reviews showing that social media marketing, branding, and proximity marketing all enhance the attractiveness of tobacco products and promote use [49, 50]. At the same time, some evidence suggests that certain e-cigarette advertisements can shape youths’ perceptions of smoking harms, in some cases reducing perceived risks [51]. Recognizing the powerful role of marketing in shaping smoking-related attitudes and behaviors is therefore essential for designing effective public health interventions and regulatory policies to curb youth initiation and ongoing use.

The multivariate analysis did not reveal a significant association between enjoyability, exposure to secondhand smoke in school buildings or at home, and smoking perceptions. Evidence from theory of planned behavior studies suggests that the lack of significance of enjoyment aligns with evidence showing that hedonic expectations tend to fade over time once social-contextual factors are accounted for [52]. Qualitative syntheses further emphasize that social belonging and stress reduction in social settings, rather than pure enjoyment, are key determinants of attitudes toward smoking [53]. Despite exposure, youth smoking perceptions did not correlate with school or home secondhand smoke exposure. However, evidence showed that smoking appeal was less associated with the presence of secondhand smoking at home [54].

Taken together, our adjusted results showed that youth e-cigarette users who perceived smoking as attractive were influenced more by feelings of comfort, targeted marketing, and gender-related factors than by general media or other exposures. These findings suggest prevention strategies should move beyond broad messaging and instead directly challenge the belief that smoking enhances social ease, tightly regulate promotional tactics that appeal to youth, and pay particular attention to the heightened vulnerability of young teenage girls.

### Strength and limitations of the study

Our study examined covariates of smoking perceptions among youth e-cigarette users, focusing on demographic, perceived enjoyment, media influence, marketing, and exposure to public places. Several limitations may affect the generalizability of these findings to other demographic groups and/or settings. First, youth may lack the cognitive maturity to accurately report their behaviors and perceptions, and self-reported data on sensitive behaviors are vulnerable to recall and social desirability bias, potentially leading to underreporting. Second, cultural and contextual factors specific to this setting may limit applicability to other countries and communities. Third, restricting the sample to intermediate-school students means the data may not capture the smoking patterns, perceptions, and experiences of youth in high schools, vocational programs, or out-of-school settings, further constraining external validity.

Other limitations are related to both the study design and the statistical modelling. The GYTS is a robust tool for monitoring youth tobacco use. However, while its cross-sectional design can identify association and generate hypotheses, it cannot provide causal understanding, let alone prove causality. This snapshot approach inherently restricts causal inference and limits the extent of which findings can inform the direction of effects, policy or intervention design. The statistical models may not fully adjust for all relevant confounders and unmeasured factors could influence both the perceived appeal of smoking and the characteristics of the youth included in the sample, potentially biasing the observed associations.

### Conclusion, implications, and recommendations

In conclusion, our findings suggest that among youth e-cigarette users, the attractiveness of smoking is shaped more by social comfort and direct promotional activities than by broader environmental or general media exposure. Although more male youth e-cigarette users rated smoking favorably, they had lower adjusted odds of viewing it as attractive than females, emphasizing the need for gender-responsive prevention strategies. Youth who perceived smoking as socially comfortable, those offered free tobacco products, or owned branded items were more likely to regard smoking as attractive, while exposure at school or home showed no independent association. Public health prevention efforts may therefore benefit from targeting comfort-based norms and industry promotional strategies. Future research should investigate these factors longitudinally and across settings to delineate causal pathways and inform tailored policy and communication approaches.

## Data Availability

Publicly available datasets were analyzed in this study. The 2022 GYTS dataset for Saudi Arabia is available via the WHO NCD Microdata Repository: Home/Central Data Catalog/GYTS/SAU_2022_GYTS_V01.

## References

[1] Lorenzo‐Blanco EI Schwartz SJ Unger JB Zamboanga BL Des Rosiers SE Huang S Latino/a youth intentions to smoke cigarettes: exploring the roles of culture and gender. J Lat Psychol (2015) 3:129–42. 10.1037/lat0000034 28042523 PMC5201205

[2] Mbongwe B Tapera R Phaladze N Lord A Zetola NM . Predictors of smoking among primary and secondary school students in Botswana. Plos One (2017) 12:e0175640. 10.1371/journal.pone.0175640 28414757 PMC5393585

[3] Garrett BE Gardiner PS Wright LTC Pechacek TF . The African American youth smoking experience: an overview. Nicotine Tob Res (2016) 18:S11–5. 10.1093/ntr/ntv203 26980860 PMC5104347

[4] Struik L Christianson K Khan S Yang Y Werstuik S-T Dow-Fleisner S Factors that influence decision-making among youth who vape and youth who don’t vape. Addict Behav Rep (2023) 18:100509. 10.1016/j.abrep.2023.100509 37519860 PMC10382621

[5] Vickerman KA Carpenter K Raskob MK Nash C Vargas-Belcher RA Beebe LA . Vaping and E-Cigarettes within the evolving tobacco quitline landscape. Am J Prev Med (2021) 60:S142–53. 10.1016/j.amepre.2020.07.013 33663702

[6] Kelly BC Vuolo M Maggs JL Staff J . E-Cigarette use among early adolescent tobacco cigarette smokers: testing the disruption and entrenchment hypotheses in two longitudinal cohorts. Tob Control (2023) 33:497–502. 10.1136/tc-2022-057717 37072167 PMC12220595

[7] Chen-Sankey J Kong G Choi K . Perceived ease of flavored E-Cigarette use and E-Cigarette use progression among youth never tobacco users. Plos One (2019) 14:e0212353. 10.1371/journal.pone.0212353 30811486 PMC6392261

[8] Murthy V . E-Cigarette use among youth and young adults. Jama Pediatr (2017) 171:209–10. 10.1001/jamapediatrics.2016.4662 27928577

[9] Ambrose BK Rostron BL Johnson SE Portnoy DB Apelberg BJ Kaufman AR Perceptions of the relative harm of cigarettes and E-Cigarettes among U.S. youth. Am J Prev Med (2014) 47: S53–60. 10.1016/j.amepre.2014.04.016 25044196 PMC4642861

[10] Hammond D Reid JL Burkhalter R Rynard VL . E-Cigarette marketing regulations and youth vaping: cross-sectional surveys, 2017–2019. Pediatrics (2020) 146. 10.1542/peds.2019-4020 32601126 PMC7329261

[11] East K Hitchman SC Bakolis I Williams S Cheeseman H Arnott D The association between smoking and electronic cigarette use in a cohort of young people. J Adolesc Health (2018) 62:539–47. 10.1016/j.jadohealth.2017.11.301 29499983 PMC5938086

[12] Staff J Kelly BC Maggs JL Vuolo M . Adolescent electronic cigarette use and tobacco smoking in the millennium cohort study. Addiction (2022) 117:484–94. 10.1111/add.15645 34286880

[13] Shahab L Beard E Brown J . Association of initial e-cigarette and other tobacco product use with subsequent cigarette smoking in adolescents: a cross-sectional, matched control study. Tob Control (2021) 30:212–20. 10.1136/tobaccocontrol-2019-055283 32184339 PMC7907552

[14] Green MJ Gray L Sweeting H . Youth vaping and smoking and parental vaping: a panel survey. BMC Public Health (2020) 20: 1111. 10.1186/s12889-020-09228-w 32718309 PMC7385857

[15] Aldukhail SK El Desouky ED Monshi SS Al‐Zalabani AH Alanazi AM El Dalatony MM Electronic Cigarette Use Among Adolescents in Saudi Arabia: A National Study, 2022. Tob Induc Dis (2025) 23. 10.18332/tid/197410 PMC11748200 39839675

[16] Smith MJ MacKintosh AM Ford A Hilton S . Youth’s engagement and perceptions of disposable E-Cigarettes: a UK focus group study. BMJ Open (2023) 13:e068466. 10.1136/bmjopen-2022-068466 36948552 PMC10040067

[17] Alshahrani NZ Alarifi AM Algethami MR Aljunaid MA Shukri A Alzain MA . Sex-stratified analysis of marketing exposure and current E-Cigarette use among saudi adolescents. Front Public Health (2025) 13:1649537. 10.3389/fpubh.2025.1649537 40832023 PMC12358419

[18] Organization WH , Prevention C for DC. Tobacco Questions for Surveys of Youth (TQS-Youth): A Subset of Key Questions from the Global Youth Tobacco Survey (GYTS) (2019).

[19] Psonka Y Vannimenus C . Perception of ENDS among French high school students in nord and pas-de-calais. Rev Mal Respir (2023) 40:743–50. 10.1016/j.rmr.2023.08.001 37633810

[20] Czarna A Szmajke A . Can cigarette smoking make a man appear sexier and stronger to women? Psychol Stud (2011) 49: 23–40.

[21] Beech JR Whittaker J . What is the female image projected by smoking? Psychologia (2001) 44:230–6. 10.2117/psysoc.2001.230

[22] Chung SS Joung KH . Risk factors for current smoking among American and South Korean adolescents, 2005-2011. J Nurs Scholarsh Off Publ Sigma Theta Tau Int Honor Soc Nurs (2014) 46:408–15. 10.1111/jnu.12099 25224519

[23] Popova L Owusu D Weaver SR Kemp CB Mertz CK Pechacek TF Affect, risk perception, and the use of cigarettes and e-cigarettes: a population study of U.S. adults. BMC Public Health (2018) 18:395. 10.1186/s12889-018-5306-z 29566752 PMC5863900

[24] Klein H Sterk CE Elifson KW . Smoke and mirrors: the perceived benefits of continued tobacco use among current smokers. Health Psychol Res (2014) 2:1519. 10.4081/hpr.2014.1519 26973934 PMC4768546

[25] Rayes BT Alalwan A AbuDujain NM Darraj A Alammar MA Jradi H . Prevalence, trends, and harm perception associated with E-Cigarettes and vaping among adolescents in Saudi Arabia. Arch Clin Biomed Res (2023) 7:147–56. 10.26502/acbr.50170327 37008304 PMC10062399

[26] Hagen EH Garfield MJ Sullivan RJ . The low prevalence of female smoking in the developing world: gender inequality or maternal adaptations for fetal protection? Evol Med Public Health (2016) 2016:195–211. 10.1093/emph/eow013 27193200 PMC4931906

[27] Smith PH Bessette A Weinberger AH Sheffer CE McKee SA . Sex/gender differences in smoking cessation: a review. Prev Med (2016) 92:135–40. 10.1016/j.ypmed.2016.07.013 27471021 PMC5085924

[28] Higgins ST Kurti AN Redner R White TJ Keith DR Gaalema DE Co-Occurring risk factors for current cigarette smoking in a U.S. nationally representative sample. Prev Med (2016) 92:110–7. 10.1016/j.ypmed.2016.02.025 26902875 PMC4992654

[29] Grard A Schreuders M Alves J Kinnunen JM Richter M Federico B Smoking beliefs across genders, a comparative analysis of seven European countries. BMC Public Health (2019) 19:1321. 10.1186/s12889-019-7700-6 31638938 PMC6805413

[30] Lund L Lauemøller SG Kjeld SG Andersen A Bast LS . Gender differences in attitudes towards a school-based smoking prevention intervention. Scand J Public Health (2020) 49:511–8. 10.1177/1403494820953325 32883175

[31] Grube JW Rokeach M Getzlaf SB . Adolescents’ value images of smokers, ex-smokers, and nonsmokers. Addict Behav (1990) 15:81–8. 10.1016/0306-4603(90)90010-U 2316415

[32] Green HD Horta M de la Haye K Tucker JS Kennedy DR Pollard M . Peer influence and selection processes in adolescent smoking behavior: a comparative study. Nicotine Tob Res (2013) 15:534–41. 10.1093/ntr/nts191 22944605 PMC3612003

[33] Jiang N Lee YO Ling PM . Young adult social smokers: their co-use of tobacco and alcohol, tobacco-related attitudes, and quitting efforts. Prev Med (2014) 69:166–71. 10.1016/j.ypmed.2014.09.013 25280439 PMC4312182

[34] Bernat D Gasquet N Wilson KO Porter L Choi K . Electronic cigarette harm and benefit perceptions and use among youth. Am J Prev Med (2018) 55:361–7. 10.1016/j.amepre.2018.04.043 30031636 PMC6168072

[35] Bobo FT Thanasekaran P Joice AJR Yadecha B Alebel A . Susceptibility to cigarette smoking and associated factors among high school students in Western Ethiopia. BMC Res Notes (2018) 11:1–5. 10.1186/s13104-018-3734-6 30165886 PMC6117939

[36] Bang I Jeong Y-J Park Y-Y Moon N-Y Lee J Jeon T-H . Secondhand smoking is associated with poor mental health in Korean adolescents. Tohoku J Exp Med (2017) 242:317–26. 10.1620/tjem.242.317 28867706

[37] Lan R Li X Chen X Hu J Luo W Lv L Secondhand smoke, genetic susceptibility, and incident chronic kidney disease in never smokers: a prospective study of a selected population from the UK biobank. Tob Induc Dis (2023) 21:58. 10.18332/tid/162607 37181462 PMC10170651

[38] Jhee JH Joo YS Kee YK Jung S-Y Park S Yoon C-Y Secondhand smoke and CKD. Clin J Am Soc Nephrol CJASN (2019) 14:515–22. 10.2215/CJN.09540818 30846462 PMC6450336

[39] Reed RM Dransfield MT Eberlein M Miller M Netzer G Pavlovich M Gender differences in first and secondhand smoke exposure, spirometric lung function and cardiometabolic health in the old order amish: a novel population without female smoking. PLoS ONE. (2017) 12:e0174354. 10.1371/journal.pone.0174354 28362870 PMC5375129

[40] Gim W Yoo J-H Shin J-Y Goo A-J . Relationship between secondhand smoking with depressive symptom and suicidal ideation in Korean non-smoker adults: the Korean national health and nutrition examination survey 2010–2012. Korean J Fam Med (2016) 37:97–104. 10.4082/kjfm.2016.37.2.97 27073608 PMC4826998

[41] Akhtar PC Currie DB Currie CE Haw SJ . Changes in child exposure to environmental tobacco smoke (CHETS) study after implementation of smoke-free legislation in Scotland: national cross sectional survey. BMJ (2007) 335:545. 10.1136/bmj.39311.550197 17827487 PMC1976539

[42] Yang T Abdullah AS Li L Rockett IRH Lin Y Ying J Public place smoke-free regulations, secondhand smoke exposure and related beliefs, awareness, attitudes, and practices among Chinese urban residents. Int J Environ Res Public Health (2013) 10:2370–83. 10.3390/ijerph10062370 23749054 PMC3717741

[43] Mantey DS Cooper MR Clendennen SL Pasch KE Perry CL . E-Cigarette marketing exposure is associated with E-Cigarette use among US youth. J Adolesc Health Off Publ Soc Adolesc Med (2016) 58:686–90. 10.1016/j.jadohealth.2016.03.003 27080732 PMC4900536

[44] Cruz TB McConnell R Low BW Unger JB Pentz MA Urman R Tobacco marketing and subsequent use of cigarettes, E-Cigarettes, and hookah in adolescents. Nicotine Tob Res Off J Soc Res Nicotine Tob (2019) 21:926–32. 10.1093/ntr/nty107 29846704 PMC6588392

[45] Pierce JP Sargent JD White MM Borek N Portnoy DB Green VR Receptivity to tobacco advertising and susceptibility to tobacco products. Pediatrics (2017) 139:e20163353. 10.1542/peds.2016-3353 28562266 PMC5470502

[46] Padon AA Lochbuehler K Maloney EK Cappella JN . A randomized trial of the effect of youth appealing E-Cigarette advertising on susceptibility to use E-Cigarettes among youth. Nicotine tob res off. J Soc Res Nicotine Tob (2018) 20:954–61. 10.1093/ntr/ntx155 29106669 PMC6037103

[47] Collins L Glasser AM Abudayyeh H Pearson JL Villanti AC . E-Cigarette marketing and communication: how E-Cigarette companies market E-Cigarettes and the public engages with E-cigarette information. Nicotine Tob Res (2018) 21:14–24. 10.1093/ntr/ntx284 29315420 PMC6610165

[48] Hines D Saris RN Throckmorton-Belzer L . Cigarette smoking in popular films: does it increase viewers’ likelihood to Smoke?1. J Appl Soc Psychol (2000) 30:2246–69. 10.1111/j.1559-1816.2000.tb02435.x

[49] Tattan‐Birch H East K Cox S Jackson SE Brown J Simonavičius E Impact of standardising the colour and branding of vape devices on product appeal among young people: a randomised experiment in England, Canada and the United States. Tob Control (2025), tc- 2024-059210. 10.1136/tc-2024-059210 40393723 PMC12353783

[50] Chacón L Mitchell G Golder S . The commercial promotion of electronic cigarettes on social media and its influence on positive perceptions of vaping and vaping behaviours in anglophone countries: a scoping review. Plos Glob Public Health (2024) 4:e0002736. 10.1371/journal.pgph.0002736 38232105 PMC10793929

[51] Vasiljevic M St John Wallis A Codling S Couturier D-L Sutton S Marteau TM . E-cigarette adverts and children’s perceptions of tobacco smoking harms: an experimental study and meta-analysis. BMJ Open (2018) 8:e020247. 10.1136/bmjopen-2017-020247 30012646 PMC6082488

[52] Barakat M Abuarab R Alkharabsheh B Budair N Fareed M Kharabsheh R Exploratory Validation of a Survey Instrument Based on the Theory of Planned Behavior to Assess Vaping Attitude and Perceptions (2024). 10.1101/2024.09.04.24313091

[53] Yan R Liu Y Huang L Li Y Huang Y Tong J Susceptibility to E-Cigarette adoption among tobacco-naïve youths: a cross-sectional study in shenzhen, China. Front Public Health (2024) 12:1320863. 10.3389/fpubh.2024.1320863 38818444 PMC11137244

[54] Kang M Lee H Jo M . Analysis of multidimensional factors in attempts to quit using tobacco by Korean adolescents. Environ Health Prev Med (2020) 25:71. 10.1186/s12199-020-00913-1 33218297 PMC7679987

